# Anti-Interleukin-1 Agents in Adult Onset Still's Disease

**DOI:** 10.1155/2012/317820

**Published:** 2012-04-29

**Authors:** Cecilia Giampietro, Bruno Fautrel

**Affiliations:** ^1^Department of Internal Medicine, University of L'Aquila, L'Aquila, Italy; ^2^School of Medicine, University Paris 6-Pierre et Marie Curie, 75013 Paris, France; ^3^Department of Rheumatology, AP-HP, Pitié-Salpêtrière University Hospital, 75013 Paris, France

## Abstract

Interleukin 1**β** (IL-1**β**) is emerging as a master mediator of adult onset Still's disease (AOSD) pathogenesis. This pleiotropic cytokine, whose expression is under the control of the inflammasome pathway, has a wide type of effects. As a key mediator of innate immunity is a potent pyrogen and facilitates neutrophilic proliferation and diapedesis into the inflamed tissues, which are key AOSD manifestations. The study of proinflammatory cytokines profiles in sera and pathological tissues of AOSD patients has shown elevated levels of IL-1**β**, these levels being highly correlated with disease activity and severity. These experimental evidences and the analogy with other autoinflammatory diseases that share with AOSD clinical and biological characteristics have suggested the blockade of IL-1**β** as a possible new therapeutic option for the AOSD, especially in conventional therapy resistant cases. Anakinra, the first anti-IL-1 agent put on the market, has demonstrated capable to induce a rapid response sustained over time, especially in systemic forms, where anti-TNF**α** failed to control symptoms. While a growing number of evidences supports the utilisation of anakinra in AOSD, a new generation of anti-IL1**β** antagonists is developing. Canakinumab and rilonacept, thanks to their higher affinity and longer half-life, could improve the management of this invalidating disease.

## 1. Introduction

Adult-onset Still's disease (AOSD) is a systemic autoinflammatory disorder characterized by daily high-spiking fevers, evanescent maculopapular rash, sore throat, polyarthritis, myalgia, lymphadenopathy, and hepatosplenomegaly [1, 2]. The etiology of AOSD is currently unknown. However, a growing number of experimental evidences supports the hypothesis that a disregulation of inflammasome complex and a related overproduction of the proinflammatory cytokine interleukin 1*β* (IL-1*β*) is a pivotal event in the pathogenesis of this disorder, in analogy with other autoinflammatory diseases that share with AOSD clinical and biological characteristics [3].

In this paper, we discuss the biology and the role of IL-1 in AOSD pathogenesis and we review the current literature about the utilization of anti-IL-1 agents in clinical practice.

## 2. IL-1 Biology

### 2.1. IL-1 Expression: The Inflammasome Pathway of Activation

IL-1*β* is a pleiotropic mediator of the response to infection and injury, which affects nearly all cell types, either alone or in combination with other cytokines. The main source of IL-1*β* is the monocytic-macrophagic system.

**Figure 1:**
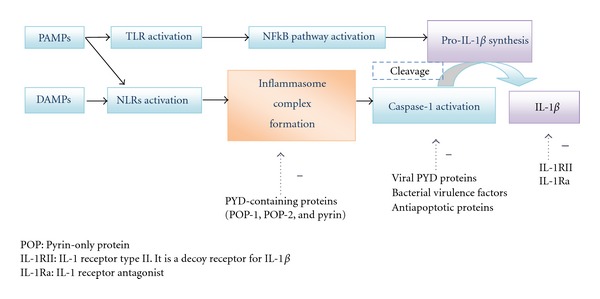
The inflammasome pathway of IL-1*β* activation.

The components of the latter do not express constitutively this protein whose availability is over the control of the innate immunity which acts as a sophisticated system for detecting signals of “danger” such as pathogenetic microbes or host-derived signals of cellular stress. Recognition occurs through a limited group of pathogen-recognition receptors (PRRs) like the Toll-like receptors (TLRs) and the NOD-like receptors (NLRs). It is the crosstalk between TLRs and NLRs that generate IL1*β* [4]. For instance, the interaction of TLR with its ligand triggers pro-IL1*β* gene expression and synthesis. The conversion of pro-IL1*β* into active IL1*β* is mediated by caspase 1 which requires NLR activation and subsequent inflammasome complex organization to exert its action. Once secreted, IL-1*β* binds and activates the single transmembrane domain type I IL-1 receptor (IL-1RI) on a variety of cell types. The following step is the recruitment of IL-1 receptor accessory protein (IL-1RAP) to the IL-1RI/IL-1*β* complex. The signal pathway initiated leads ultimately to nuclear factor *κ*B (NF*κ*B) translocation in the nucleus and finally to the expression of an array of inflammatory genes [5–7] (Figure 1).

### 2.2. An Alternative Pathway of Activation

Interestingly, recent advances suggest that caspase-1 may not be the only responsible of IL-1*β* activation. Neutrophil serine proteases, mast cell chymase, and metalloproteinases may also be able to proteolytically cleaving pro-IL-1*β* [8, 9]. This may account for the articular damage occurring in AOSD.

### 2.3. IL-1 Effects

#### 2.3.1. Cytokine Production and Development of Autoinflammation

**Figure 2:**
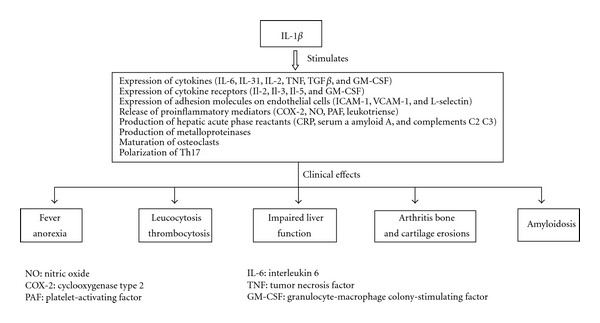
IL-1*β* actions.

IL-1*β* upregulates cytokines, acute phase proteins, and tissue remodeling enzymes. As a key mediator of innate immunity is a potent pyrogen and facilitates neutrophilic proliferation and diapedesis into inflamed tissues, which are key AOSD manifestations. In the periphery, IL-1*β*, in synergy with TNF, is recognised as an important factor in driving bone and cartilage erosion. Of more, it increases platelet production, which results in thrombocytosis, and promotes the production of IL-6, which in turns stimulates hepatocytes to synthesize several acute phase proteins [10, 11] (Figure 2).

#### 2.3.2. Orientation of the Immune Response

IL-1*β* also plays an important role in the adaptive immune response acting directly on naïve and memory T cells to promote their expansion and survival and instructing B cells to enhance antibody production. Moreover, it has recently been discovered that IL-1 drives the development of T_H_17 cells, which are now known to be a key T-cell subset mediating many autoimmune and chronic inflammatory diseases. The frequency of circulating Th17 cells is elevated and positively correlated with disease activity in AOSD patients [12–16].

### 2.4. IL-1 Regulation

As it plays a critical role in host defence eliciting a wide range of effects, the activity of IL-1*β* needs to be tighltly controlled to avoid tissue damage resulting in autoreactive response. The regulation takes place on several levels, including transcription, mRNA stability, translation, maturation, secretion, receptor expression, and release of soluble receptors, as well as IL-1RII, a decoy receptor and IL-1Ra, a naturally expressed inhibitor of IL-1 receptor occupancy [17, 18]. The complexity of IL-1*β* biology explains well why the aberrant activation of the innate immunity system can lead to a multitude of chronic diseases, like AOSD (Figure 1).

## 3. IL-1*β* in AOSD Pathogenesis

AOSD is a member of the expanding group of the “autoinflammatory disorders” [19].

In contrast with autoimmunitary diseases that are primarily caused by dysregulation of adaptive immune responses, autoinflammatory diseases are caused by disorders in the innate inflammatory pathways and the usual hallmarks of autoimmunity such as high titers of antigen-specific T lymphocytes and autoantibodies are characteristically absent [20].

One of the major events in the pathogenesis in these syndromes is an increased release of active IL-1*β*. Cytokine profile in AOSD sera is characterized by the presence of IL-6, IFN*γ*, IL-18, and, of note, IL-1*β* [21, 22].

The identification of the critical role of NLRP3 inflammasome in the maturation of IL-1*β* motivated the study of its role in the pathogenesis of autoinflammatory syndromes. Mutations of the inflammasome related genes have been identified in the cryopyrin-associated periodic syndromes (CAPSs), in familial Mediterranean fever (FMF) and in the pyogenic arthritis, pyoderma gangrenosum, and acne syndrome (PAPA). Conversely, in AOSD the cause of innate immunity disarray still remains unknown.

The* primum movens* of the pathogenetic sequence seems to be an infectious agent, as suggested by the temporal relationship between disease onset and viral syndromes. Other pathogenic organisms (bacteria, parasites, or chemical events) may also be involved. This hypothesis is consistent with a role of the innate immunity in AOSD. Anyway, infection alone is unlike to be sufficient to trigger AOSD, and a predisposing genetic background is probably required even if no consistent associations with HLA aplotypes have been individuated [23]. Youm et al. have studied 83 AOSD patients to investigate whether IL-1*β* and IL-1Ra gene polymorphisms are associated with the development and clinical features of AOSD, but no differences were observed between patients and healthy controls [24]. A potent IL-1*β* production-inducer is IL-18, a member of the IL-1 family that growing evidences are demonstrating to be pivotal in promoting the systemic inflammatory process of AOSD. IL-18 is important in both innate and acquired immune responses. It exerts pleiotropic effects such as the stimulation of neutrophil migration and activation, the polarization of T-cell response versus the Th1 phenotype, the expression of adhesion molecules and the activation of natural killer cells [25]. It synergizes with IL-6 in the production of ferritin from macrophage-lineage cells [26] and with IL-23 in the induction of Th17 cells [16]. IL-18 serum levels are higher in AOSD patients compared to other autoinflammatory diseases [27] and correlate with disease activity, serum ferritin levels, and neutrophil count. The local expression of IL-18 may be responsible for tissue damage in some target organs such liver [28] and synovial membranes [29].

However, actually the most convincing case supporting the central role of IL-1*β* in AOSD pathogenesis is the dramatic and sustained efficacy of IL1-blockade on AOSD symptoms, even in refractory forms of the disease.

## 4. IL-1-Targeting Therapies

### 4.1. Anakinra

Anakinra (Kineret) is the first IL-1 inhibitor designed. It is a recombinant, nonglycosylated form of human IL-1 receptor that acts as a pure receptor antagonist binding tightly to the IL-1RI and preventing activation of this receptor by either IL-1*β* or IL-1*α*. It is administered subcutaneously once daily, due to its short half-life. Approved by the FDA in 2001 for treating patients with rheumatoid arthritis, its use validated the importance of IL-1 in a broad spectrum of inflammatory diseases, AOSD included. A growing number of reports describe a rapid response to anakinra characterized by impressive reduction in disease activity, fever resolution, and normalization of hematologic and biochemical parameters within hours to days after the first injection in patients affected from AOSD refractory to other synthetic or biological treatment [3, 22, 30–34]. These effects are likely to be sustained over a long treatment period and allow tapering and withdrawal of concomitant glucocorticoids and DMARDs and finally anakinra administration in monotherapy [34–36]. Such a long-lasting effect was not described with TNF-blockers, whose efficacy seems to decrease over time [37]. Our group has observed that in some patients that achieved a complete and stable remission was possible not only a discontinuation or reduction of treatment associated, but also of anakinra itself, without relapse [38]. These data seem to be confirmed by the retrospective study of Laskari et al. [36]. The possibility of dose reduction may enhance compliance and drug adherence and highlights the potential cost-effectiveness of anakinra. Moreover, according to our observations, the most impressive results were obtained in patients with systemic forms. This is consistent with data reported by Lequerré et al. [34]. We can argue that the proinflammatory cytokine IL-1 could have a more prominent role in systemic forms of AOSD for whom Anakinra use can be reserved. In these cases, Anakinra may be efficacious not only to induce remission, but also to prevent new relapses in polycyclic forms and the amyloidosis development associated with long-standing elevated inflammatory markers. Methotrexate and TNF-blockers may remain interesting in chronic articular presentation of AOSD [39].

Another open question is whether anakinra must be introduced early in the course of AOSD or only after other conventional treatment failure to avoid the adverse effects of a prolonged corticotherapy and to limit the social impact of a poorly controlled disease [2]. To support the necessity of an earlier introduction of anakinra, Moulis et al. have reported two cases of dramatic side effects secondary to conventional treatment that later had an immediate and remarkable response after anakinra starting [40].

Immunomodulation by anti-IL1 is rather safe and well tolerated. The most frequent adverse event reported in the clinical trials is injection-site reaction which is generally mild to moderate and rapidly resolutive. Some infections have been reported; however, it has not been associated with the development of tuberculosis or other fungal infections, demyelinating syndromes or worsening of congestive heart failure [41]. A recent systematic review of literature conducted by Singh et al. on 163 randomized controlled trials with 50,010 participants and 46 extension studies with 11,954 participants that included indirect comparisons between anakinra and other biological agents revealed that anakinra is associated with a significantly lower risk of serious adverse events compared to most other biologic treatments [42]. Anecdotally, we report a severe systemic inflammatory response syndrome after anakinra introduction [43] and an episode of thrombocytopenia, which appeared one week after anakinra introduction and resolved soon after its discontinuation [44].

Immunization against anakinra has been described, but the appearance of potentially neutralizing antibodies is transient and has never been associated with anakinra failure subsequently do not preventing chronical anakinra administration [45].

In conclusion, even if more studies are needed, all the evidences actually available strongly support the importance of anakinra as new safe and effective therapeutic option in AOSD treatment.

### 4.2. Next Generation Anti-IL-1*β* Antagonists

Although anakinra has been demonstrated effective and safe, its short half-life demanding daily injections often associated with painful local adverse reactions limits its usefulness in the clinical practice. This has represented an incentive to the development of new anti-IL1*β* antagonists with different pharmacokinetic properties. Recently, soluble receptors for IL-1 (rilonacept) and human mAbs to IL-1*β* (canakinumab and gevokizumab) have been used to neutralize IL-1*β* specifically (Table 1).

*Rilonacept* (Regeneron) is a construct of 2 extracellular chains of the IL-1R complex (IL-1R plus IL-1RAP) fused to the Fc portion of human IgG1. Since it contains both receptor components, rilonacept is able to bind IL-1*β* and IL-1*α* with high affinity. It has an half-life is longer that  anakinra, and it is administrated once weekly [46].

 Henderson et al. investigated the use of rilonacept in a small cohort of refractory AOSD patients observing a good clinical and biological response. Interestingly, they reported that high levels of IL-18 at baseline were predictors of a successful response. This evidence suggests this biomarker as a useful tool to predict response to treatment [47].

*Canakinumab* (Ilaris) is a human monoclonal IgG1 antibody targeted against IL-1*β*, thus preventing its binding to the receptor complex. Generated by a transgenic mouse strain, it offers a high specificity for IL-1*β*. Among IL-1 antagonists, canakinumab is the agent with the longest half-life. Of more, it has been demonstrated that its clinical efficacy is prolonged beyond what expected based on its half-life. Subsequently, pharmacokinetic modelings have shown a positive feedback of IL-1*β* on its own production. This explains why the effectiveness of the drug is greater than its half-life, thus permitting its administration once every two months [48, 49]. In clinical trials, canakinumab and rilonacept are associated with a mild increase of infections. The rate of injection site injections was 34% versus 27% in rilonacept versus placebo and 9% versus 14% in canakinumab versus placebo [50, 51].

 Actually, canakinumab and rilonacept are indicated only for the treatment of the CAPS, but they could represent a new therapeutic option for AOSD [52, 53].

Gevokizumab (XOMA 052) is an IgG2-humanized mAb against human IL-1*β*. XOMA has completed a successful proof-of-concept Phase 2 trial of gevokizumab in patients with Behçet's uveitis, types 1 and 2 diabetes, and rheumatoid arthritis. It belongs the potential to treat patients with a wide variety of inflammatory diseases, including autoinflammatory diseases [54, 55].

The development of IL-1*β* blockers with higher affinity and longer half-life could improve patient management and also favor patient compliance.

## Figures and Tables

**Table 1:** Anti-IL1 agents.

Biologic agent target cytokine(s)	Molecular characteristics	Doses (route of administration)	Recorded indications
Anakinra (Kineret) IL-1*α* and IL-1*β*	Recombinant human IL-1 receptor antagonist	100 mg/day (sc)	Rheumatoid arthritis
Rilonacept (Regeneron) IL-1*α* and IL-1*β*	Fusion protein of the extracellular domains of IL-1R1 and IL-1RAP, coupled to the Fc region of human IgG	Induction dose: 320 mg maintenance: 160 mg/week (sc)	Cryopyrin-associated periodic syndromes (CAPSs)
Canakinumab (Ilaris) IL-1*β*	Fully human monoclonal anti-IL1*β* antibody	150 mg every 8 weeks (sc)	Cryopirin-associated periodic syndromes (CAPSs)
Gevokizumab (Xoma 052) IL-1*β*	Recombinant humanized anti-IL1*β* antibody		Not labeled potential therapeutic use: Behçet's uveitis, types 1 and 2 diabetes, and rheumatoid arthritis (phase II trials)

sc: subcutaneous.
